# Acetylation-defective mutants of Pparγ are associated with decreased lipid synthesis in breast cancer cells

**DOI:** 10.18632/oncotarget.2371

**Published:** 2014-08-19

**Authors:** Lifeng Tian, Chenguang Wang, Fred K. Hagen, Michael Gormley, Sankar Addya, Raymond Soccio, Mathew C. Casimiro, Jie Zhou, Michael J. Powell, Ping Xu, Haiteng Deng, Anthony A. Sauve, Richard G. Pestell

**Affiliations:** ^1^ Department of Cancer Biology, Sidney Kimmel Cancer Center, Thomas Jefferson University, Philadelphia, PA, USA; ^2^ Department of Biochemistry and Biophysics, University of Rochester, Rochester, NY, USA; ^3^ Division of Endocrinology, Diabetes, and Metabolism, Department of Medicine, Department of Genetics, and The Institute for Diabetes, Obesity, and Metabolism, Perelman School of Medicine at the University of Pennsylvania, Philadelphia, PA, USA; ^4^ Department of Pharmacology, Weill Medical College of Cornell University, York Avenue LC216, New York, NY, USA; ^5^ Proteomics Resource Center, Rockefeller University, New York, NY, USA

**Keywords:** acetylation, peroxisome proliferator-activated receptor gamma (Pparγ), nuclear receptors, silent mating type information regulation 2 homolog 1 (SIRT1), lipogenesis

## Abstract

In our prior publications we characterized a conserved acetylation motif (K(R)xxKK) of evolutionarily related nuclear receptors. Recent reports showed that peroxisome proliferator activated receptor gamma (PPARγ) deacetylation by SIRT1 is involved in delaying cellular senescence and maintaining the brown remodeling of white adipose tissue. However, it still remains unknown whether lysyl residues 154 and 155 (K154/155) of the conserved acetylation motif (RIHKK) in Pparγ1 are acetylated. Herein, we demonstrate that Pparγ1 is acetylated and regulated by both endogenous TSA-sensitive and NAD-dependent deacetylases. Acetylation of lysine 154 was identified by mass spectrometry (MS) while deacetylation of lysine 155 by SIRT1 was confirmed by *in vitro* deacetylation assay. An *in vivo* labeling assay revealed K154/K155 as bona fide acetylation sites. The conserved acetylation sites of Pparγ1 and the catalytic domain of SIRT1 are both required for the interaction between Pparγ1 and SIRT1. Sirt1 and Pparγ1 converge to govern lipid metabolism *in vivo.* Acetylation-defective mutants of Pparγ1 were associated with reduced lipid synthesis in ErbB2 overexpressing breast cancer cells. Together, these results suggest that the conserved lysyl residues K154/K155 of Pparγ1 are acetylated and play an important role in lipid synthesis in ErbB2-positive breast cancer cells.

## INTRODUCTION

Peroxisome proliferator-activated receptor gamma (PPARγ) is a member of the nuclear receptor (NR) superfamily, which functions as a ligand-dependent transcription regulator. Due to alternative splicing and differential promoter utilization, PPARγ exists in two isoforms, PPARγ1 and PPARγ2. The murine Pparγ2 encodes an additional 30 amino acids (28 amino acids in human PPARγ) at its N-terminus. PPARγ1 is expressed in many tissues at low levels, while PPARγ2 is expressed at high levels and is restricted to adipose tissue. Mouse PPARγ1 and human PPARγ1 shared 98% homology in protein sequence. PPAR regulates diverse biological functions including adipocyte differentiation [1], lipogenesis [2], inflammation [3], insulin sensitivity [4], cellular proliferation [5], and autophagy [6]. Both natural ligands including prostaglandins (15d-PGJ2) and synthetic ligands including the anti-diabetic thiazolidinediones (TZD) are known to induce PPARγ activity.

PPARγ activity is also regulated by post-translational modifications, including phosphorylation, sumoylation, ubiquitination and acetylation. Among these post-translational modifications, phosphorylation has been extensively studied. The Activation Function 1 (AF1) region of PPARγ is phosphorylated by mitogen-activated protein kinases (MAPKs) (PPARγ1 at Ser82 and PPARγ2 at Ser112), which represses transcriptional activity by inhibiting ligand binding and altering cofactor recruitment [7-9]. Phosphorylation of the same residue by cyclin-dependent kinases, Cdk7 and Cdk9, promotes PPARγ activity [10, 11]. Recently it was reported that PPARγ2 is phosphorylated at Ser273 by cyclin-dependent kinase 5 (CDK5) [12]. Phosphorylation of PPARγ2 by CDK5 inversely correlates with TZD-induced insulin sensitivity in human. These studies indicate that phosphorylation of PPARγ at different sites or even at the same sites results in different transcriptional and functional outcomes depending on the physiological context and the kinases involved. This phenomenon is well known among other post-translationally modified nuclear receptors. Several different acetylation sites (K266, 268, 302, and 303) of estrogen receptor alpha (ERα) have been reported [13, 14]. Acetylation of ERα at lysine 266 and 268 enhances the DNA binding and transactivation activities of the receptor, however acetylation of lysine 302 and 303 suppresses the transactivation function. Recent publications showed that PPARγ is acetylated by p300 or CBP and deacetylated by silent mating type information regulation 2 homolog 1 (SIRT1). PPARγ acetylation participated in cellular senescence [15] and the brown remodeling of white adipose tissue (Lysine 268 and 293) [16]. Even though acetylation has been well characterized in the androgen receptor (AR) and ERα at a conserved lysine motif (K(R)xxKK) which is shared amongst evolutionarily related nuclear receptors [14, 17-19], the function of this conserved lysine motif in Pparγ was not known.

Increased *de novo* fatty acid synthesis, which contributes to energy homeostasis and tumor growth, is a common feature of human tumors. The survival of breast cancer cells, especially those with ErbB2/Her2 overexpression, is highly dependent upon the lipid metabolism induced by Pparγ, which protects cells from palmitate toxicity [20]. Our previously published work showed that a constitutively active Pparγ1 mutant (PγCA) collaborated with oncogenic ErbB2 to promote mammary tumor growth [21].

Herein, we characterized Pparγ1 acetylation at the conserved lysine motif (RIHKK). Our studies revealed that Pparγ1 is acetylated at nine distinct lysyl residues. The acetylation of Pparγ1 lysine 154 was confirmed by a chemically modified trypsin mapping protocol developed for histone acetylation mapping. The K155 residue, which is located in close proximity to the DNA binding domain, was deacetylated by SIRT1. The Pparγ1 mutant K154/155R reduced the acetylation levels assessed by *in vivo* labeling. The acetylation-defective mutant K154/155A or K154/155Q showed reduced interaction with SIRT1. The Pparγ1 K154/155 determines the induction of lipogenesis in ErbB2 overexpressing breast cancer cells. Loss of SIRT1 function and gain of Pparγ1 function converge on common gene signaling pathways. In summary, these results suggest that the acetylation of the conserved lysine motif (K154/155) of Pparγ1 determines lipid synthesis in ErbB2-positive breast cancer cells.

## RESULTS

### Acetylation of lysyl residues of conserved motif in Pparγ

**Figure 1:**
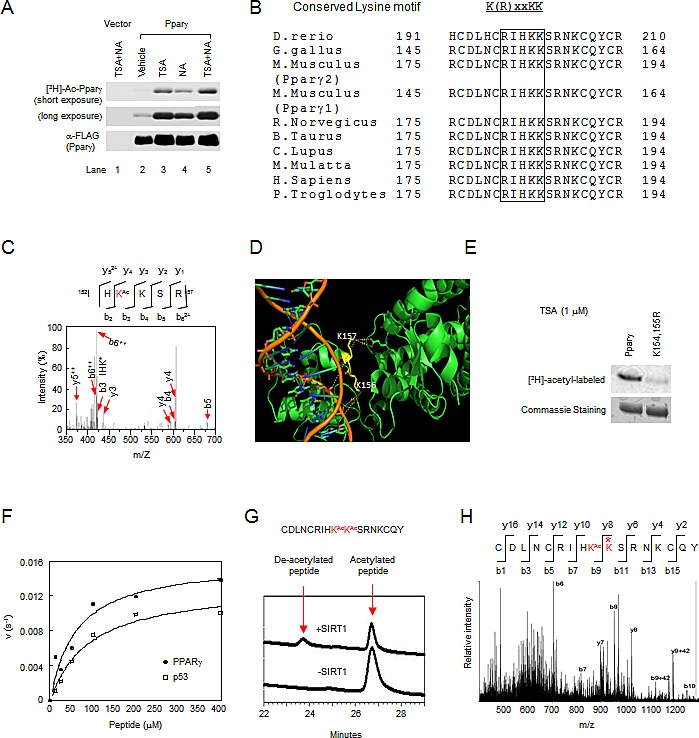
Acetylation of lysyl residues within conserved acetylation motif of Pparγ (A) The inhibition of either TSA-sensitive or NAD-dependent deacetylase activity induced Pparγ acetylation assayed by *in vivo* [^3^H]-sodium acetate labeling. (B) The conserved acetylation motif in Pparγ is shown for several species. (C) Tandem MS (MS/MS) spectrum of Pparγ showing the acetylation at K154 (Red letter), “Ac” indicates lysine (K) residues that is acetylated. (D) A cylinder model for the crystal structure of hPPARγ1 with DNA double helix (orange color). The lysine 156 and 157 are shown in yellow. (E) Mutant K154/155R significantly reduced Pparγ acetylation. (F) The deacetylation rate of Pparγ or p53 peptide of different concentrations by SIRT1. The lines are best fits to the Michaelis-Menten equation and determine the following values for *K*_m_ and *V*_max:_ PPARγ (•), *Km* is 60.1 μM, *vmax* is 0.016s-1; ASp53 (ƀ), *Km* is 90.5 μM, *vmax* is 0.013s-1. (G) HPLC chromatograms of Pparγ peptide deacetylation with SIRT1 enzyme. Untreated diacetylated Pparγ peptide is shown at bottom chromatogram. (H) MS/MS spectrum of the deacetylated Pparγ. Asterisk indicated deacetylated lysine residue.

Pparγ acetylation has historically been investigated by using anti-acetyl lysine antibodies [15, 16]. In order to determine Pparγ acetylation, we firstly performed *in vivo* labeling assays. The incorporation of [^3^H] acetyl-CoA into Pparγ was only seen in cells transfected with 3xFLAG-tagged Pparγ1, but not control vector (Fig. 1A). The addition of trichostatin A (TSA), an inhibitor of type I/II HDACs, or nicotinamide (NA), an inhibitor of Sirtuins, increased [^3^H]-labeled Pparγ (Fig. 1A). These results suggest that the basal acetylation levels of Pparγ are very low, and endogenous class I/II and III HDACs are both involved in the deacetylation of Pparγ.

The residues in proximity to Pparγ1 K154/155 resemble the acetylated motif of ERα and AR [14, 17] and are conserved among different species (Fig. 1B). In order to determine whether the lysyl residues K154 and K155 in the conserved motif of Pparγ are acetylated, we conducted mass spectrometry (MS) of immune-precipitated 3xFLAG-tagged Pparγ1 from HEK 293 cells. These lysyl residues contain numerous basic amino acids which are often resistant to conventional trypsin mapping. This was confirmed by trypsin and chymotrypsin mapping (Fig. S1A), which demonstrated that mapping covered 92% of the protein, but omitted the conserved acetylation motif. Therefore, trypsin mapping was conducted using a chemical-derivatization protocol [22]. While the sequence coverage (Fig. S1B) is not as high as conventional trypsin or chymotrypsin mapping, the spectral counts and Mascot scores for acetylated peptides were higher, and multiple peptides with overlapping sequences mapped to the same site (Fig. S2A, B). A total of nine lysyl residues including K154 were identified as targets for acetylation *in vivo* (Fig. 1C and Fig. S2A). Using 3-dimensional structural mapping, lysyl residues 156 and 157 of human PPARγ1 (lysyl residues 154 and 155 in mouse Pparγ) are located in the DNA binding domain in close juxtaposition to the ligand binding domain (Fig. 1D). The side chain nitrogen of the K156 (K154 in mouse, bottom yellow residue) is approximately 8A° from the phosphate backbone of DNA, too far for a hydrogen bond, and therefore does not directly contact DNA (orange in image) but rather appears solvent accessible. This data is consistent with its observed acetylation by MS. The adjacent K157 (K155 in mouse, top yellow residue) side chain nitrogen is approximately 13A° from the DNA backbone, forms a hydrogen bond with E378 of human PPARγ but appears solvent inaccessible. To verify the acetylation of the conserved lysine motif in Pparγ, an *in vivo* labeling assay was performed in HEK 293 cells transfected with 3xFLAG-tagged Pparγ1 wild-type or K154/155R mutant. Wild-type Pparγ1 was acetylated while the K154/155R mutation significantly reduced the incorporation of [^3^H] acetyl-CoA (Fig. 1E), suggesting that K154/155 are bona fide acetylation sites.

As NA increased the Pparγ acetylation, we investigated whether Pparγ1 K154/155 could serve as a substrate for SIRT1. A synthetic peptide containing the sites was used as a substrate for an *in vitro* deacetylation assay. The kinetic parameters for deacetylation of this peptide were similar to that of a p53 peptide known to be deacetylated by SIRT1 (Fig. 1F). Confirmation of the peptide deacetylation (K155) was obtained by MS (Fig. 1G, H). Thus, consistent with our original findings [13, 17] in which the lysyl residues of a conserved motif (K(R)xxKK) in ERα and AR are acetylated, and subsequent studies of other nuclear receptors (NRs) [18, 19, 23-25], Pparγ1 is also acetylated at the conserved lysine motif.

### Mutation of Pparγ1 at K154/155 reduces SIRT1 binding

**Figure 2:**
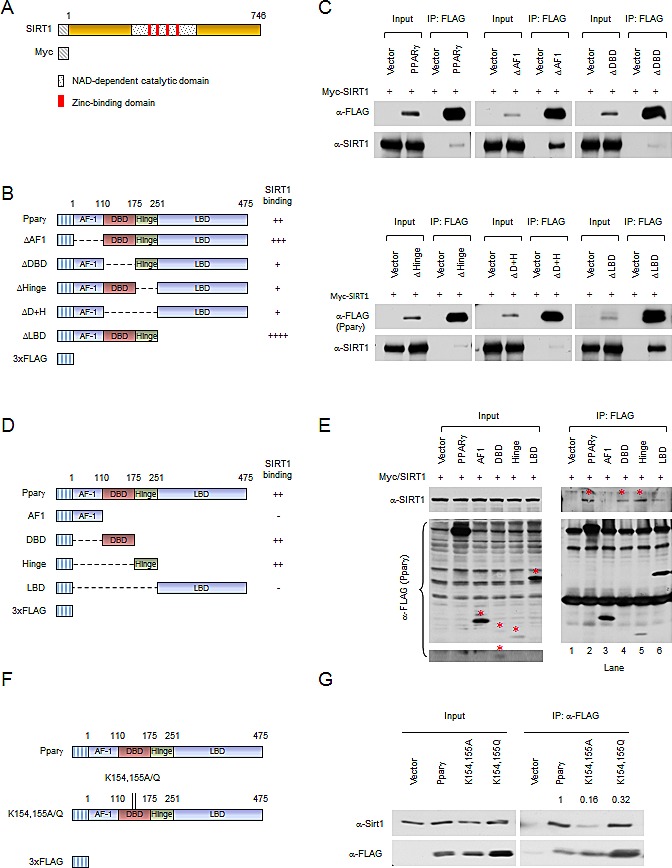
Mutation of Pparγ at K154/155 reduces SIRT1 binding (A) Schematic diagram of Myc-tagged SIRT1 wild-type. (B) Schematic diagrams of 3xFLAG-tagged Pparγ1 full length and internal deletion mutants. (C) HEK 293T cells were transfected with the indicated plasmids. Immuno-precipitations with anti-FLAG antibody were conducted, and Western blot was performed by indicated antibodies. The abundance of SIRT1, PPARγ internal deletions in input are shown. (D) Schematic diagrams of 3xFLAG-tagged Pparγ1 full length and individual domains. (E) HEK 293T cells were transfected with the indicated plasmids. Immuno-precipitations with anti-FLAG antibody were conducted, and Western blot was performed by indicated antibody. The bands for 3x FLAG-tagged Pparγ full length and individual domains are indicated by an asterisk. (F) Schematic diagrams of 3xFLAG-tagged Pparγ1, Myc-tagged SIRT1 wild-type and catalytic point mutation. (G) HEK 293T cells were transfected with the indicated plasmids. Immuno-precipitations with anti-FLAG antibody were conducted, and Western blot was performed by indicated antibody. All experiments were performed at least 3 times, representative figures are shown.

The association between Pparγ1 and SIRT1 was next examined by immune-precipitation and Western blot analyses. We firstly examined the domains of Pparγ1 required for SIRT1 binding. Expression vectors encoding Myc-tagged SIRT1 (Fig. 2A) together with FLAG-tagged Pparγ1 or internal deletion mutants (Fig. 2B) were transiently introduced into HEK 293T cells. Immune-precipitation was performed. Protein expression was evidenced by Western blot of the input protein using an anti-FLAG antibody for Pparγ1 and mutants, and an anti-Myc antibody for SIRT1 (Fig. 2C). SIRT1 was detected in immune-precipitated wild-type Pparγ1. Deletion of the AF-1 domain or ligand binding domain (LBD) of Pparγ1 enhanced relative SIRT1 binding. Deletion of the DNA binding domain (DBD) and/or hinge region reduced binding (Fig. 2C). Given that deletion of the DBD and/or hinge region reduced SIRT1 binding, we determined whether SIRT1 is associated with the individual DBD or hinge domains. Expression vectors encoding individual Pparγ1 domains (Fig. 2D) were co-expressed with SIRT1 expression vector in HEK 293T cells. Western blot with anti-Myc antibody showed either the DBD or hinge region of Pparγ1 was sufficient for association with SIRT1. The relative binding of SIRT1 to the AF1 region and LBD was reduced compared to wild-type Pparγ1 (Fig. 2E). We further studied the interaction between the Pparγ1 K154/155 mutation and SIRT1. As shown in Figure 2F and 2G, both K154/155A and K154/155Q demonstrate reduced binding to SIRT1. These results suggest that the DBD and/or hinge regions of Pparγ1 are required for SIRT1 association and that both the K154/155A and the K154/155Q mutants have reduced SIRT1 binding ability.

### SIRT1 deletion and Pparγ gain-of-function govern common signaling pathways *in vivo*

**Figure 3:**
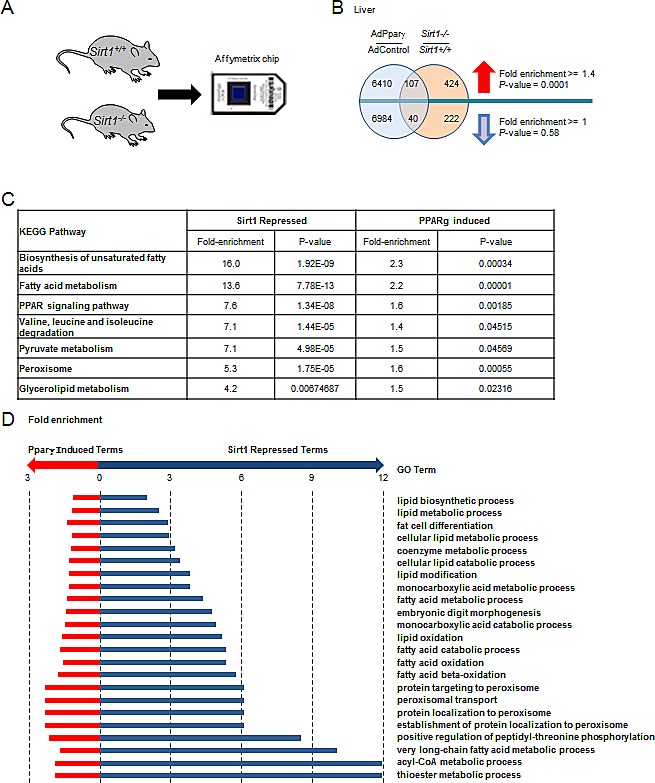
Sirt1 and Pparγ are both required for maintaining essential metabolic pathways in the liver (A) Schematic representation of microarray screening of deferentially expressed genes between *Sirt1*−/− mouse liver and *Sirt1*+/+ mouse liver. (B) Overlap of genes regulated by Pparγ overexpression and Sirt1 knockout in mouse liver. (C) KEGG pathways enriched for genes up-regulated in Pparγ overexpressing and Sirt1 knockout liver. (D). Gene Ontology Biological Processes enriched for genes up regulated in Pparγ overexpressing and Sirt1 knockout liver. Hypergeometric test used to identify deregulated ontologies associated with Pparγ overexpression and Sirt1 knockout. Fold induction ≥ 1.5 and *P*-value ≤ 0.05.

Given that Pparγ enhances adipogenesis [26] and Sirt1 attenuates adipogenesis [27], we investigated the possibility that SIRT1 deletion and Pparγ gain-of-function may have common gene expression signatures *in vivo*. Total RNA from liver samples was isolated from *Sirt1^−/−^* mice and littermate controls [28]. Microarray analysis identified 262 genes that were significantly down-regulated and 531 genes that were significantly increased in *Sirt1^−/−^* mouse liver relative to *Sirt1^+/+^* mouse liver (*P* < 0.05, Fold > 1.5) (Fig. 3A, B and Table S1). Gene network analysis populated lipid metabolism as the major downstream target of Sirt1 (Tables S2). For the gene expression profile of Ppar gain-of-function, we took advantage of published data conducted in mouse liver injected with an adenovirus encoding mPparγ1 [29]. The hepatic genes altered in *Sirt1^−/−^* mice were compared to those regulated by Pparγ1 overexpression. A significant number of genes (107 genes with 76 expected giving a fold enrichment of 1.4 and a *P*-value of 0.0001) were up-regulated by Pparγ1 overexpression and Sirt1 gene deletion (Fig. 3B). Seven KEGG pathways and 23 Biological Process Gene Ontologies were enriched for genes up-regulated by Pparγ1 overexpression and Sirt1 gene deletion (Fig. 3C, D). Such pathways were mainly involved in lipid metabolism, including the biosynthesis of unsaturated fatty acids, fatty acid metabolism, and fatty acid oxidation. These data suggested SIRT1 and Pparγ converge to govern lipid metabolism *in vivo.*

### The conserved acetylation sites of Pparγ determine lipid production

**Figure 4:**
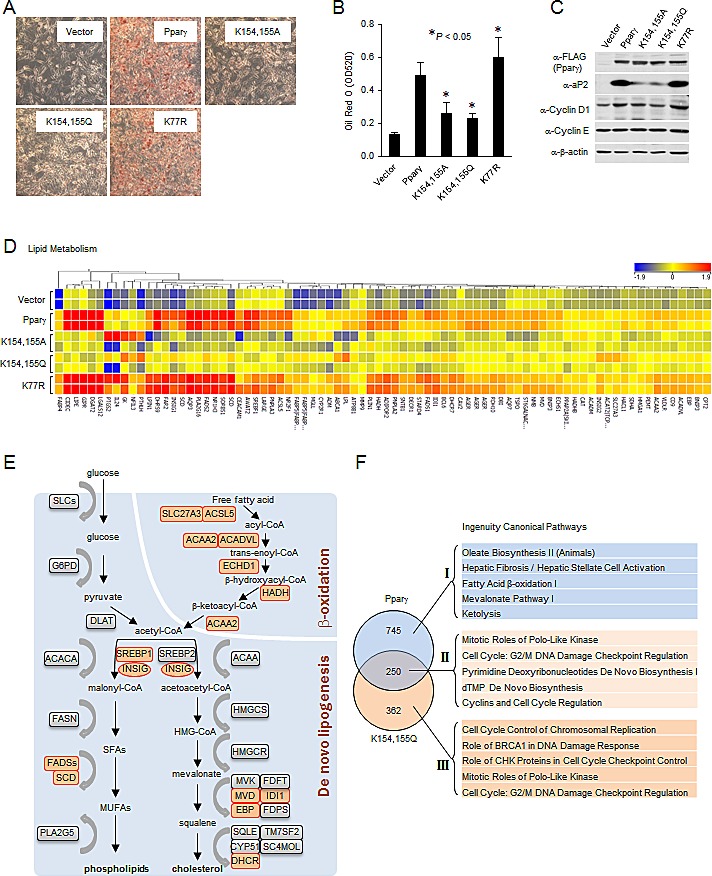
Acetylation-defective mutant of Pparγ is associated with decreased lipogenesis (A) MCF10A-NeuT cells expressing various mutants of Pparγ were cultured until they were confluent, after 2 days, cells were stained with Oil Red O, and photographed. (B) Quantitative analysis of lipids in cells shown in (A) was performed by measuring the OD 520 nm of the Oil Red O stained cells eluted with 4% Igepal CA-40 in isopropanol (v/v). The results are shown as the average of three experiments; the bars indicate mean ± SEM. (C) Total cellular proteins from cells subjected to the same experimental protocol as in (A) were collected, and analyzed by Western blot as indicated. (D) Heat map of genes of lipid metabolism differentially regulated by Pparγ and vector control. (E) Schematic representation of the key enzymes of lipogenic signaling pathway. Genes upregulated by Pparγ are highlighted (orange color). (F) The genes differentially regulated in MCF10A-NeuT cells expressing wild-type Pparγ or Pparγ acetylation mutant were analyzed by the Ingenuity Pathway. The top five Canonical Pathways were listed corresponding to the loss of function with Pparγ acetylation mutant (I), the gain of function (III) and those unchanged by acetylation (II).

Given Sirt1 deacetylates Pparγ and inhibits adipogenesis, we reasoned that acetylation of Pparγ may promote lipid synthesis. In order to investigate the role of the conserved acetylation sites of Pparγ in lipogenesis, ErbB2 overexpressing breast cancer cells were used and lysyl residues were substituted with alanine (K to A) or glutamine (K to Q) to generate residues that were incapable of being acetylated. Substitution mutations (K154/155A, K154/155Q and K154/155R) of Pparγ1 K154/155 were generated, and transduced into MCF10A-NeuT cells. K77R of Pparγ1, a Pparγ mutant that is defective in SUMOylation [30, 31], was used as a positive control since this mutant induces adipogenesis in NIH3T3 cells. Following a differentiation protocol, Oil Red O staining followed by subsequent quantitative measurement was used to examine the lipid accumulation. Pparγ induced lipid accumulation which was further enhanced by Pparγ1 K77R (Fig. 4A). In contrast, the Pparγ1 K154/155A and the K154/155Q mutants were defective in the induction of adipogenic differentiation and lipid accumulation (Fig. 4A, B). Consistent with the ability of Pparγ to induce lipid formation, the relative protein abundance of the adipocyte Protein 2 (aP2) was induced by Pparγ1 and the Pparγ1 K77R mutant but not the K154/155A or K154/155Q mutants (Fig. 4C). Under differentiation conditions, the expression levels of the cell cycle regulators, cyclin D1 and cyclin E, were slightly increased or unchanged respectively (Fig. 4C). Next, in order to study the gene expression profile regulated by Pparγ acetylation, microarray analysis was performed. Genome-wide expression analysis identified 995 genes differentially expressed in Pparγ1-transduced MCF10A-NeuT cells (Tables S3). Among these, pathway analysis identified 127 genes populating the enhanced lipid metabolism pathway (Fig. 4D and Tables S4). Key enzymes required for *de novo* lipogenesis and *β*-oxidation are upregulated (highlighted by orange color) by wild-type Pparγ, but not K154/155A or K154/155Q substitution mutants (Fig. 4E). Comparison of the gene expression pathways regulated by Pparγ vs. the K154/155Q (Fig. 4F) showed distinct pathways dependent upon the K154/155 with the loss-of-function (Fig. 4F, I and Tables S5) or gain-of-function (Fig. 4F, III and Tables S6). Unchanged pathways are shown in Tables S7. These data suggest that substitution of lysyl residues with residues that cannot be acetylated (alanine (K to A) or glutamine (K to Q)) represents PPARγ acetylation-defective mutant in and that Pparγ acetylation at the conserved lysine motif serves as a molecular switch of Pparγ-mediated induction of lipogenesis in breast cancer cells.

## DISCUSSION

In this report, we have shown that the conserved acetylation site (K154/155) of Pparγ plays a critical role in breast cancer cell lipid synthesis. Firstly, we identified nine distinct acetylation sites in cultured cells by MS, and further confirmed the acetylation of K154/155 of Pparγ by *in vivo* labeling assay. We analyzed the function of K154/155 acetylation based on the conservation of this motif across species and evolutionally related nuclear receptors [14]. Our results showed that acetylation-defective mutants of Pparγ are associated with decreased lipogenic differentiation in ErbB2-positive breast cancer cells, as shown by Oil Red O staining, protein expression of the classic adipocyte marker aP2, and mRNA expression of multiple lipogenic genes in microarray analysis. Decreased lipogenic function of the K154/155A and K154/155Q mutant was not due to changes of protein structure, cellular localization, and protein stability (unpublished data). This work provides a novel mechanism through which Pparγ regulates lipid metabolism via conserved acetylation sites in breast cancer cells.

Acetylation is a dynamic post-translational modification of lysyl residues. Han *et al.* reported that acetylation and deacetylation of Pparγ is regulated by p300 and SIRT1 in the process of cell senescence [15], however no acetylated residues were identified. Recent publication by Li *et al*. mapped 5 acetylated lysyl residues (98, 107, 218, 268, and 293) in mouse Pparγ2 (68, 77, 188, 238 and 263 in mPparγ1) by MS [16]. Among them, two evolutionally conserved residues in the helix 2-helix region, Lys268 and Lys293 were further investigated. These studies indicated that deacetylation of Pparγ at Lys268 and Lys293 by SIRT1 is required for maintaining the brown remodeling of white adipose tissue [16]. However, these prior studies did not analyze acetylation of the conserved lysine motif (K(R)xxKK) in Pparγ due to the limited coverage of the MS protocol used. Similar to the N-terminus of histone, the region “RIHKKSRNKC” is enriched with an overwhelming number of arginine and lysyl residues. Small fragments obtained by conventional trypsin digestion cannot be detected by conventional MALDI-TOF. Therefore, in our studies, trypsin mapping after a chemical-derivatization protocol was used to render lysine positions resistant to trypsin cleavage. This method increased the spectral counts and Mascot scores of acetylated peptides. A total of nine lysyl residues including K154 were identified. Among them, K188 and K238 in Pparγ1 correspond to K218 and K268 in Pparγ2, which were reported by Li *et al* [16]. Discrepancies in the identified Pparγ acetylation sites may be the results of two different experimental approaches and/or the choice of Pparγ isoform choose in these two studies. In our approach, we transfected HEK 293 cells with mPparγ1, and treated with TSA and NA, while in their report, HEK 293 cells were co-transfected with expression vectors encoding mPparγ2 and acetyltransferase Cbp, followed by treatment with Rosiglitazone (a PPARγ agonist). Quantification of K154/155 acetylation was performed. The results suggest that the native acetylation levels of Pparγ are very low (1%), consistent with Li *et al* [16] in which the acetylation of mPparγ2 K268 and K293 was detected by MS only in the presence of Cbp. Acetylation of K155 was not detected by MS, most likely due to its inaccessibility by solvent as shown by a 3-dimensional structure analysis. Using an *in vitro* deacetylation assay, we demonstrated that a Pparγ peptide harboring acetylated K154 and K155 residues is a substrate for SIRT1 deacetylation. Finally, in our *in vivo* labeling studies, the Pparγ mutant K154/155R conveyed significantly reduced incorporation of [^3^H] acetyl-CoA. Together, these results demonstrate that K154 and 155 are bona fide acetylation sites *in vivo* and are substrates for SIRT1 deacetylation.

Han *et al.* reported the catalytic domain of SIRT1 is necessary and sufficient for the interaction between Pparγ and SIRT1 and that the catalytically inactive SIRT1 mutant H363Y conveyed reduced association with Pparγ [15]. In our studies, the DBD and/or hinge regions of Pparγ were required for SIRT1 association. The K154/155A or K154/155Q mutants showed reduced association with SIRT1. These data are consistent with our MS data, where all nine acetylation lysyl residues identified by MS are located in the DNA binding domain and hinge domain of Pparγ. Our results indicate that SIRT1 binds and deacetylates Pparγ. This led us to investigate the pathways regulated by SIRT1 and Pparγ *in vivo*. As expected, a significant number of genes (107 genes) were up-regulated by Pparγ overexpression and Sirt1 gene deletion. KEGG pathways and Biological Process Gene Ontologies populated lipid metabolism as the major term, including the biosynthesis of unsaturated fatty acids, fatty acid metabolism, and fatty acid oxidation. Recent studies have shown that the function of SIRT1 in metabolic homeostasis requires NAD+-dependent deacetylase activity. Sterol regulatory element binding protein (SREBP) family proteins are critical regulators of lipogenesis and cholesterologenesis. Walker, *et al*. and Ponugoti, *et al*. showed that SIRT1 can directly deacetylate SREBPs, and that SIRT1 activity is important in the fasting-dependent attenuation of SREBP function [32, 33]. Several other nuclear receptors regulated by acetylation and deacetylation are also involved in lipid metabolism [34, 35]. Li, *et al.* reported that SIRT1 directly deacetylates LXRs, resulting in increased LXRs turnover and enhanced target gene expression [34]. LXRs are nuclear receptors that function as cholesterol sensors and regulate whole body cholesterol and lipid homeostasis. SIRT1 also regulates bile acid homeostasis through direct deacetylation of FXR. Down-regulation of hepatic SIRT1 increases FXR acetylation with deleterious metabolic outcomes [35]. More recently, Li *et al*. showed that a gain-of-function SIRT1 promotes “browning” of WAT by deacetylating Pparγ at Lys268 and Lys293 [16]. Herein, we showed that loss-of-function of SIRT1 and gain-of-function of Pparγ converge on liver lipid metabolism. The interaction between Pparγ and SIRT1 potentially controls the acetylation and deacetylation status of Pparγ protein. Therefore, deacetylation of Pparγ by SIRT1 could serve as a molecular switch that acts as key metabolic sensor.

Enhanced lipogenesis is a hallmark of cancer cells [36]. This especially holds true in ErbB2-positive human breast cancer cells, which have a high degree of fat storage [37]. We have previously shown that active Pparγ promotes ErbB2-positive breast cancer growth through enhanced angiogenesis [21]. In the current study, we investigated the role of Pparγ K154/155 acetylation on lipid production in ErbB2-positive breast cancer cells. Adipogenic differentiation assays were performed to evaluate the function of these mutants in MCF10A cells transformed with oncogene NeuT. Consistent with data from Yamashita *et al*., Pparγ2 induced adipogenesis in NIH3T3 cells and a sumoylation-defective K107R mutant of Pparγ2 stimulates adipogenesis more robustly than the wild-type [31]. We showed that Pparγ induced lipid accumulation in MCF10A-NeuT cells, and the K77R mutant of Pparγ1 increased lipogenesis. Oil Red O staining, lipogenic protein expression and genome-wide expression analysis indicated that both K154/155A and K154/155Q mutants were defective in the induction of lipid accumulation and adipogenic differentiation. As shown in Figure 4, key enzymes and transcription factors required for *de novo* lipogenesis (SREBP, Insig and SCD) and *β*-oxidation (ACAA and MCD) are up-regulated by Pparγ wild-type, but not K154/155A or K154/155Q mutants. K154/155R mutant exhibited similar effect to Pparγ wild type (data not shown). In our prior publication, we showed that ERα acetylation governs ligand sensitivity, as all substitution mutants (K to A, Q and R) induced ERα hormone sensitivity [14]. Recently, Daniel *et al*. identified the progesterone receptor (PR) acetylation site within the conserved lysine motif KxKK (amino acids 638–641) [18]. Mutation of these three lysyl residues to alanine (A) or glutamine (Q) resulted in delayed phosphorylation, nuclear entry and transactivation of c-Myc, a known rapid response gene. In summary, SIRT1 deacetylates Pparγ at a conserved lysine motif. SIRT1 deletion and Pparγ gain-of-function converge to govern lipid metabolism *in vivo*. We conclude that acetylation of Pparγ increases lipid synthesis. These data suggest that the Pparγ acetylation of the lysine motif serves as a molecular switch governing Pparγ-mediated induction of lipogenesis in ErbB2/Her2 overexpressing breast cancer cells. It is of great importance to further investigate if lipogenesis regulated by acetylation of the Pparγ lysine motif (K154/155) contributes to the progression of ErbB2-positive breast cancer.

## MATERIALS AND METHODS

### Cell culture, plasmid DNA, and transfection

The HEK293, HEK293T cells were maintained in Dulbecco's Modification of Eagle's Medium (DMEM) supplemented with 10% fetal bovine serum, 1% penicillin, and 1% streptomycin. MCF10A-NeuT cells were cultured as previously described [38]. MCF10A-NeuT cells transduced with Pparγ1 or mutants were maintained in Dulbecco's Modification of Eagle's Medium (DMEM) supplemented with 10% fetal bovine serum, 1% penicillin, and 1% streptomycin. All cells were cultured in humidified atmosphere with 5% CO_2_ at 37°C. Rosiglitazone and 15d-PGJ2 are from Cayman Chemical.

The expression vectors encoding mouse Pparγ1 are previously described [39]. The Pparγ1 point mutants were derived by site-directed mutagenesis using sequence-specific primers. The wild-type and point mutants of Pparγ1 were individually cloned into p3xFLAG-CMV 10 vector (Sigma) and MSCV-IRES-GFP vector. The internal deletion mutants of Pparγ1 were subcloned into p3xFLAG-CMV 10 vector using sequence-specific primers. The individual domain of Pparγ1 was digested by *XbaI/BamHI* from GAL4-DBD-HA-Pparγ1-AF1, DBD, Hinge and LBD (Gifts from Dr. Z. [40]), and then was subcloned into p3xFLAG-CMV 10 vector. The integrity of all constructs was confirmed by sequence analysis. The Myc-tagged wild-type and mutant (H363Y) SIRT1 expression constructs in pcDNA3.1 were previously described [41].

Cell transfection and infection were performed as previously described [42, 43]. Retroviruses were prepared by transient cotransfection of vector expressing Pparγ1, mutants or empty vector together with the helper viral vector into 293T cells using calcium phosphate precipitation. The retroviral supernatants were harvested 48 h after transfection and filtered through a 0.45 μm filter. Mammary epithelial cells MCF10A-NeuT cells were incubated with fresh retroviral supernatants in the presence of 8 μg/ml polybrene for 24 hrs, cultured for a further 48 hrs, and subjected to different assays.

### *In vivo* [^3^H]-acetyl-CoA labeling

Labeling of Pparγ was conducted using the previously described protocol with some modifications [44]. Briefly, one 10-cm plate of 293 cells was used for *in vivo* acetyl group labeling. The cells transfected either with expression vector or control were maintained in DMEM w/ 10% FBS at over 80% confluence. The cells were first were treated with 1μM TSA and 10 mM NA for 4 hours, and then transferred to DMEM medium containing 1μM TSA, 10 μM NA and 1 mCi/ml [^3^H]-sodium acetate (75–150 mCi/mmol) (Perkin Elmer) for 1hr. Cells were washed twice with ice-cold PBS and lysed in RIPA buffer with freshly added protease inhibitor cocktail supplemented with fresh DTT (1 mM) and PMSF (1 mM). The lysates were centrifuged at 14,000 rpm for 15 min at 4°C. Supernatants were incubated with antibody conjugated to agarose beads, for 6 to 12 hrs at 4°C. Immunopurified proteins were resolved on SDS–PAGE gels. Gels were either staining with Coomassie blue and then dried, or directly dried and subjected to autoradiography at -70°C for 2-4 weeks.

### Identification of Pparγ acetylated lysine residue using MS

*Preparation and proteolytic digestion of Pparγ:* 3xFLAG-tagged Pparγ1 was purified by immunoprecipitation, using anti-FLAG M2 affinity gel, and the bound material was eluted using a soft SDS-elution protocol in order to selectively elute the bound recombinant protein and not the anti-FLAG antibody. This eluent was concentrated on a speedvac and further separated on SDS-PAGE and stained with Coomassie blue. The major band at 55 kDa was cut. The gel pieces were completely dehydrated and subsequently reduced and alkylated. To improve coverage of the basic regions of Pparγ1 sequences, the gel isolated protein was mapped using a modified trypsin mapping protocol as below.

*Chemical modification of lysine and tryptic mapping of acetylation site:* Chemical modification was achieved after the reduction/alkylation steps. In detail, the gel was hydrated with 10 μl of deuterated acetic anhydride, 20 μl of 100 mM ABC (pH 8), and mixing. The acetylation reaction is fast and results in acidification of the solution, so 70 μl of 100 mM ABC is immediately added and the solution is incubated at 37°C for 5 minutes. The pH is measured within the first 5 minutes and adjusted to 7-8. After an additional 30 minute incubation at 37°C, the supernatant is removed and the gel pieces are washed twice with water, and then dehydrated by successive washes (with vortexing, as above) in 100 μl of 50 mM ABC, then 100 μl 50 mM ABC with 50% acetonitrile (50-ACN), and finally 100% ACN. This chemical treatment derivatized lysyl residues with a deuterated acetyl group (a 45Dalton mass increase) and rendered lysine positions resistant to trypsin cleavage. Deuterated acetic anhydride was used to allow differentiation from the native acetyl group (a 42 Dalton modification group).

Trypsin (200 ng in 50 μl of 50 mM ABC) is then added to hydrate the gel slices. After an hour at room temperature, additional 50 mM ABC and 10% ACN was added to ensure that the gel pieces were always covered with solvent. The digest was transferred to 37°C and digested overnight. Next, the supernatant was transferred to LoBind tubes (Eppendorf) and the remaining peptides were extracted by adding sufficient 50% ACN, 5% Trifluoroacetic acid to cover the gels and by vortexing for 25 minutes. This step was repeated and each of the three supernatants were combined and lyophilized in a Speedvac.

*Mass spectrometry analysis:* Dehydrated peptides were resuspended in 5% acetonitrile, 0.05% formic acid and immediately loaded on a nano-spray tip for LC-MS/MS analysis. 10 - 15% of the peptide digest is loaded on a Magic C18 AQ (Michrom) nanospray tip, packed to 5 cm. This tip was loaded, using a pressure bomb, and washed, after installation on the HPLC of a Thermo LTQ mass spectrometer, with 5% methanol, 0.1% formic acid, for 10 min with a flow rate of 600 nl/minute (about 10 column volumes = 6.6 μl) The peptide digests were analyzed in an LC-MS/MS run, using a 5-15 % methanol gradient over 2.5 minutes, followed by a 15-60% methanol gradient for 67 minutes, a 60% methanol isocratic step of 4 minutes, ending with a 3-minute 95% methanol step, with all solvents containing 0.1% formic acid. A full MS survey scan is performed every 3 seconds and the top 7 peaks are selected to produce MS/MS fragmentation spectrum. In order to confirm if K154/155 is acetylated, the same peptide digest was run a second time, under identical HPLC conditions. With the mass spectrometer programmed with an inclusion filter of 383-431 m/z to select for doubly charged peptides in the size range of the IHKKSR hexa-peptide in its unmodified and fully derivatized states. This selective method allows for the determination of the relative frequency of the modified and unmodified IHKKSR peptides, by evaluating the spectral count ratios.

*Mapping of proteolytic peptide fragments and acetylation sites:* The MS and MS/MS fragmentation spectrum data were used in a Mascot search of the whole mouse proteome. To identify peptide sequences modified with acetyl groups, a custom database, containing the recombinant Pparγ sequence, was also searched. The following search criteria were used for selecting fragmentation spectra that map to proteolytic peptides: peptide tolerance = -0.8 to +0.5, a minimum ion score of 15, and a fragmentation spectrum, containing fragment ions that either include or flank the acetylated amino acid position. Mascot searches were conducted, allowing for multiple positive charge-states, 2, 3, or 4 missed cleavage sites, fixed S-carboxyamidomethyl modification of cysteine and variable methionine oxidation and lysine acetylation. Mascot searches use trypsin digestion, but allow for 4 or 5 missed trypsin cleavages, and variable deuterated acetyl (45 Da) and native acetyl (42 Da) modification.

### Determination of the deacetylation site in Pparγ peptide *in vitro*

To determine the Michaelis-Menten curves of SIRT1-catalyzed deacetylation of Pparγ and p53, the Pparγ peptide CDLNCRIHXXSRNKCQY and the p53 peptide GSRAHSSHLXSXXGQSTSRHRXLMFXTEGPDSD where “X” is AcK (Rockefeller Proteomics Resource). The enzymatic reaction was initiated by adding SIRT1 into the solution containing the peptide. For the Pparγ peptide, the Pparγ peptide of different concentrations was incubated with 4 μM SIRT1, 500 μM NAD, and 500 μM DTT in the 100 mM KH_2_PO_4_ buffer of pH 8. The reaction was quenched with 10% TFA after 10 minutes incubation at 37^o^C. The Pparγ peptide and the deacetylated peptide were separated by HPLC (Hitachi LaChrom Elite HPLC system) using a C18 5 μm column (Waters) with gradient acetonitrile and 0.1%TFA as eluent. The areas of peaks for peptides were used for quantification. For the p53 peptide, the p53 peptide of different concentrations was incubated with 4 μM SIRT1, 500 μM NAD in 100 mM KH_2_PO_4_ buffer of pH 8 at 37^o^C. The enzymatic product acetyl adenosine diphosphate ribose (AADPR) was monitored by HPLC using C18 column (Waters) with 20 mM NH_4_OAc as eluent. The area of AADPR peak was used for quantification. The turnover rate versus the Pparγ peptide and p53 peptide concentration was fitted with Kaleida Graph software, and *K_m_* and *k*_cat_ were determined.

In order to determine the deacetylation site in the Pparγ peptide, 200 μM Pparγ was incubated with 4 μM SirT1 for 20 minutes in the reaction of 1.25 mL. The deacetylated peptide was separated from the Pparγ peptide by HPLC as previously described and collected. The lyophilized product was analyzed for mass spectrometry at the Proteomics Resource Center in the Rockefeller University. In the analysis, the samples of Pparγ and the deacetylated Pparγ were separately mixed with 50% aqueous methanol solution (vol:vol=1:1) and loaded into a glass nanospray PicoTip (New Objectives). The samples were analyzed by nano-electrospray tandem mass spectrometry with an ABI QSTAR mass spectrometer.

### Immunoprecipitation and Western blot

HEK293T or HEK293 cells were transfected with an expression vector as indicated in the figure. 24 h after transfection, the cells were harvested in cell lysis buffer (50 mM HEPES (pH 7.2), 150 mM NaCl, 1 mM EDTA, 1 mM EGTA, 1 mM DTT, 0.1% TritonX-100) supplemented with protease inhibitor mixture (Roche Diagnostics). Whole cell lysates (500 μg) were immunoprecipitated with 10μl of M2 beads (A2220; Sigma) or 2 μg of antibodies as indicated. Immunoprecipitates were washed 5 times with cell lysis buffer and resolved by SDS-PAGE gel followed by Western blot with the indicated antibodies. Antibodies used were: anti- FLAG^®^ M2 Affinity Gel (M2; Sigma), anti- PPARγ (H100 and E8), anti-SIRT1 (H-300), anti-cyclin E (M-20), anti-aP2 (C-15), anti-β-actin (C4) (Santa Cruz), and anti-cyclin D1 (DCS-6; Santa Cruz or Ab3; NeoMarker). The abundance of immunoreactive protein was quantified using a densitometer (Image Quant version 1.11, Molecular Dynamics Computing Densitometer, Sunnyvale, CA).

### Adipogenic differentiation and Oil Red O staining

MCF10A-NeuT cells expressing various mutants of Pparγ1 were maintained at confluence for 2 days and treated with vehicle control or ligand for 2 days. Oil Red O staining of cells has been previously described [43].

### Microarray analysis

Total RNA was isolated from MCF10A-NeuT cells transduced with Pparγ1 or acetylation defective mutant after adipogenic differentiation protocol. RNA quality was determined by an Agilent 2100 bioanalyzer. Probe synthesis and hybridization to Affymetrix gene chips, human gene 1.0 ST array (Affymetrix, Santa Clara, CA) were performed according to the manufacturer's manual. Chips were scanned on an Affymetrix Gene Chip Scanner 3000, using Command Console Software. Background correction and normalization were done using Iterative plier 16 with GeneSpring V12.0 software (Agilent, Palo Alto, CA, USA). 1.5-fold (*p* value <0.05) differentially expressed gene list was generated. The differentially expressed gene list was loaded into Ingenuity Pathway Analyses (IPA) 8.0 software (http://www.ingenuity.com) to perform biological network and functional analysis. Expression profiles are displayed using Treeview. Microarray analysis was also performed with total RNA samples from *Sirt1*^+/+^ and *Sirt1*^−/−^ mice liver [28]. Pathway analysis was performed using and Kyoto Encyclopedia of Genes and Genomes (KEGG). Gene Ontology (GO) analysis was performed for gene functions.

## SUPPLEMENTARY MATERIAL FIGURES


