# Supplementary material for: Acetylation-defective mutants of Pparγ are associated with decreased lipid synthesis in breast cancer cells
Source: Oncotarget. 2014 Aug 19;5(17):7303–15. doi: 10.18632/oncotarget.2371 (PMC4202124; doi:10.18632/oncotarget.2371)
Supplement: Supplementary file 1 [file oncotarget-05-7303-s001.pdf]

Acetylation-defective mutants of Ppary are associated with decreased lipid synthesis in breast cancer cells

Supplementary Material

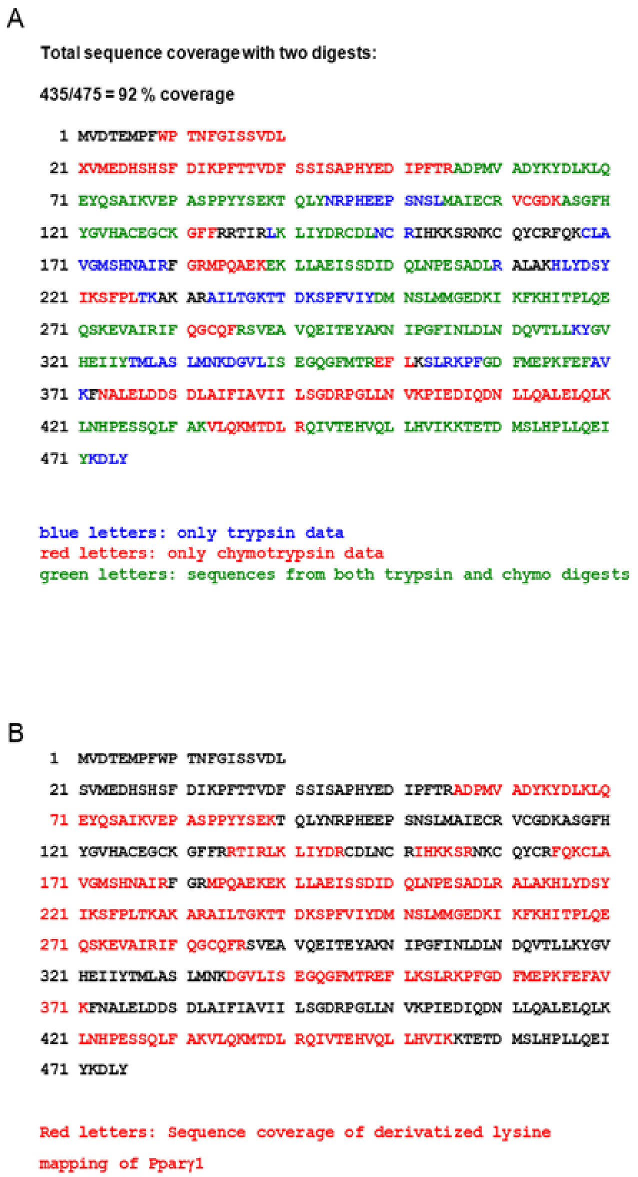

Figure S1: The sequence coverage with different digestion. Mass spectrometry was conducted of immune-precipitated Pparγ in transfected HEK293 cells. Analysis totally covered >96% of the protein. (A) Chymotrypsin and trypsin mapping and sequence coverage of Pparγ1. (B) Sequence coverage of derivatized lysine mapping of Pparγ1.

A

Acetylation position	Mascot score	Peptide sequence*
140	25.14	LK(ac)LIYDR
154	21.29	IHK(ac)K*SR
167	20.55	FQK(ac)C*LAVGMSHNAIR
167	20.53	FQK(ac)C*LAVGM*SHNAIR
167	16.84	NKC*QYC*RFQK(ac)C*LAVGMSHNAIR
188	68.96	MPQAEK(ac)EK*LLAEISSDIDQLNPESADLR
188	52.69	M*PQAEK(ac)EK*LLAEISSDIDQLNPESADLR
188	65.16	DRQAEK(ac)EKLLAEISSDIDQLNPESADLR
188	38.36	DRQAEK(ac)EK*LLAEISSDIDQLNPESADLR
188 & 190	35.42	DRQAEK(ac)EK(ac)LLAEISSDIDQLNPESADLR
222	53.46	ALAK*HLYDSYIK(ac)SFPLTK*AK*AR
228	39.70	ALAK*HLYDSYIK*SFPLTK(ac)AK*AR
238/242	45.00	AILTGK(ac)TTDK(ac)SPFVIYDMNSLMMGEDKIK

*asterisks indicate chemically-derivatized lysine positions
"ac" indicates lysine (K) residues that are acetylated

B

Position[1]	Peptide sequence[2]	Mascot score[3]	Expect value[3]	Spectral counts[4] (p<0.05)[5]	
				w/ dynamic exclusion[6]	inclusion filter[7]
K154	IHK(Ac)K*SR	21	0.015	7 (3)	5 (2)
unmodified	IHK*KSR	29	0.0041	1 (0)	25 (14)
unmodified	IHKK*SR	33	0.0035	4 (2)	16 (10)
unmodified	IHK*K*SR	37	0.0005	8 (6)	235 (225)

Notes:
1. position of native acetylated residue
2. "Ac" indicates acetylated amino acid.
3. *chemically-derivatized lysine position
4. Mascot scores and values for the top peptide hit
5. spectral counts for peptide queries with an Expect value less than 0.1
6. in parenthesis are spectral counts for peptide queries with a probability score less than 0.05
7. from LC-MS/MS runs that use dynamic exclusion to increase the diversity of the sample set
8. from LC-MS/MS runs filter peptides based on their m/z value and only conduct MS/MS fragmentation analysis on peptides with a m/z between 383 – 431.

Figure S2: Peptide mapping the basic region of Pparγ (a.a. positions 152-157). (A) Mass spectrometry identified nine acetylated lysine residues by a chemical-derivatization protocol. (B) LC-MS/MS analysis of derivatized Pparγ after trypsin digestion. The spectral counts and Mascot scores for acetylated peptides were shown.
